# Hypertonic Saline Solution Drives Neutrophil from Bystander Organ to Infectious Site in Polymicrobial Sepsis: A Cecal Ligation and Puncture Model

**DOI:** 10.1371/journal.pone.0074369

**Published:** 2013-09-17

**Authors:** Mariana Cardillo Theobaldo, Flavia Llimona, Ricardo Costa Petroni, Ester Correia Sarmento Rios, Irineu Tadeu Velasco, Francisco Garcia Soriano

**Affiliations:** Emergency of Medicine Division-Faculdade de Medicina da Universidade de São Paulo, São Paulo, Brazil; D'or Institute of Research and Education, Brazil

## Abstract

The effects of hypertonic saline solution (HSS) have been shown in several animal models of ischemia and shock. Literature has shown potential benefits of HSS modulating inflammatory response after sepsis in an animal model. We studied the HSS effects in sepsis through cecal ligation and puncture (CLP) in Balb-C mice. Groups studied: 1- CLP without treatment (CLP-C); 2- CLP treated with normal saline solution NaCl 0.9% – 34 ml/Kg (CLP-S); 3- CLP treated with HSS NaCl 7.5% – 4 ml/Kg (CLP-H); and 4- group (Basal) without no CLP or treatment. Volume infusion was always applied 30 min after CLP. Lung and peritoneal lavage were harvested after 6h and 24h of CLP to analyze cytokines amount, oxide nitric, lipid peroxidation and neutrophil infiltration. Neutrophil infiltration, ICAM-1, CXCR-2, and CXCL-1 in lung were reduced by HSS (CLP-H) compared to CLP-C or CLP-S. Neutrophil in peritoneal lavage was increased in 24h with HSS (CLP-H) compared to CLP and CLP-S. Peritoneal CXCR-2 was increased in CLP-C and CLP-S but presented a lower increase with HSS (CLP-H) after 6 hours. GRK-2 presented difference among the groups at 24 h, showing a profile similar to neutrophil infiltration. Pro-inflammatory cytokines (TNF-α and IL-6) were reduced by HSS treatment; CLP-S increased TNF-α. IL-10 was increased in lung tissue by the HSS treatment. The oxidative stress (TBARS and nitric oxide biochemistry markers) was reduced with HSS. Animal survival was 33.3% in CLP-C group, 46.6% in CLP-S group and 60% in the CLP-H group after the sixth day. The HSS protects the animal against sepsis. Our results suggest that the volume replacement modulate pro and anti-inflammatory mediators of an inflammatory response, but HSS presented a more effective and potent effect.

## Introduction

Sepsis and septic shock are characterized by an acute systemic immune response to a variety of bacterial infections. The host response could be triggered by bacteria, viruses and fungi [1]; most cases are Gram-negative bacteria (60%) and the remainder of Gram-positive [2], [3]. Host receptors recognize distinct bacterial components and initiate the signaling for the inflammatory response [4]–[7].

Septic shock is the most severe response of systemic infection and is a major cause of morbidity and mortality in non-cardiac intensive care units (ICUs) around the world [8], [9]. Approximately 25% to 35% of all septic episodes end in death [10], with high mortalities rates in both underdeveloped and developing countries [8]. In the United States approximately 750,000 patients are treated for severe sepsis yearly with a mortality rate of 30–50% and an estimated $17 billion in health care costs [9]. In Brazil, several studies already showed high mortality rates [8]. Patients suffering from septic peritonitis experience a higher mortality rate (60% to 80%) [11]. Despite advances in diagnosis, antibiotic therapy and supportive care, mortality has remained high and disproportionately affects the chronically ill and the aged [9].The exaggerated proinflammatory response during sepsis may result in many of the injurious and sometimes fatal physiological symptoms of the disease. Strategies targeting a single mediator have failed as an effective treatment in sepsis. Treatments able to modulate the amplifier aspects of inflammation will be more efficient [12]–[14]. In spite of increased knowledge on septic mechanism up to now the therapeutic used has not added improvement in survival patients [15].

The therapy consists basically in source control and hemodynamic support with volume expansion. Recently the positive volume balance has been associated to pulmonary and abdominal complications [16]. The pioneering study by Velasco *et. al.* (1980) showed that small amounts of 7.5% saline solution restored vital parameters and decreased mortality in dogs submitted to severe hemorrhagic shock [17]. Since the 1980’s, the Hypertonic Saline Solution (HSS) has been extensively studied, at a dose of 4 ml/kg into a peripheral vein [17]–[20].

Recent clinical trial showed a benefit of HSS in septic patients [21]. Experimental studies showed beneficial effects of HSS modulating inflammatory response, as for instance the expression and release of cytokines, free radicals, augmenting interleukin-10, and reducing oxidative burst [22]. Hypertonic saline solution appears as a way to modulate excessive inflammation, effective hemodynamic support with no risk for volume overload damages.

The aim of this study is to analyze the role of hypertonic saline in the inflammatory profile of sepsis and neutrophil migration into the lung and the peritoneal cavity. We studied in experimental sepsis, through model of cecal ligation and puncture (CLP), the hypertonic solution effects on inflammatory response. The model of CLP in mice is very well established and can address the objectives of our study about sepsis and the hypertonic solution treatment avoiding using dogs or rabbits.

## Methods

### Ethical approval

Procedures were performed in accordance the Guide for the Care and Use of Laboratory Animals published by the US National Institutes of Health. The study protocol was approved by the Research Ethics Committee of the USP School of Medicine (Comissão de Ética para Análise de Projetos de Pesquisa do HCFMUSP - http://www.hcnet.usp.br/adm/dc/cappesq/) (# 0333/08). For experimental procedures, animals were anesthetized with pentobarbital 20 mg/mL.

### Procedures

#### Sepsis induction – Cecal ligation and punction, and treatments

A total of 297 male BALB/c mice, 8 wk old, 25 g of mean weight, subjected to CLP, were used in this study, as described previously [23]. The animals were provided from our School Facility, they are specific pathogen free (SPF) in climatized facility, kept in automatic dark/side cycle (http://www.biot.fm.usp.br/). Under aseptic conditions, a 2 cm midline laparotomy was performed to allow exposure of the cecum with adjoining intestine. The cecum was tightly ligated with a 3.0 silk suture at its base, below the ileocecal valve, and was perforated twice with a 22-gauge needle (top and bottom). The cecum was gently squeezed to extrude a small amount of feces from the perforation sites. The cecum was then returned to the peritoneal cavity and the laparotomy was closed with 4.0 silk sutures. In addition, n =  24 animals were used for control purposes. All animals were then returned to their cages with free access to food and water.

The animals were divided into four groups: the group without treatment cecal ligation and puncture (CLP-C); septic treated with hypertonic saline solution 7.5% (4 ml/kg) (CLP-H); the third group treated with normal saline solution 0.9% (34 ml/kg) (CLP-S); and the sham group (Basal) to indicate the basal values without both CLP and treatment. The groups CLP-H and CLP-S were treated 30 min. after CLP. The volume (saline and hypertonic saline solution) was infused in the tail vein of the animals [24]. The animals were anesthetized and sacrificed to collect samples of peritoneum lavage and lung after 6h and 24h of CLP. The samples were collected by a blinded collaborator, the tissues were numbered, and the code kept with the collaborator up to the end of measurements. We used the lower number of animals necessary to each experiment, for biochemical assays we used between 6–8 animals.

#### Tissue Preparation

Frozen tissue (100 mg) was pulverized in liquid nitrogen. Samples were homogenized in NP40 buffer containing 135 mM NaCl, 20 mM Tris (pH 8,0), 10% glycerol, and proteolytic enzyme inhibitors (40 ug/mL phenylmethylsufonylfluoride 1 mM; Sigma, St, Louis, MO). After debris separation through centrifugation for 40 min at 10,000 rpm, the supernatants were collected and protein concentration was determined by the Bradford method (Bio Rad, Hercules, CA). Samples were stored at –80°C until assayed.

#### Measurement of cytokines, chemokines and adhesion molecules

The concentration of Tumor Necrosis Factor -α, Interleukin (IL) -10, IL-6, Inter-Cellular Adhesion Molecule 1 (ICAM-1), Vascular cell adhesion protein 1 (VCAM-1) and neutrophil chemoattractant chemokine (C-X-C) motif ligand 1 (CXCL-1) were measure in lung tissues by enzyme-linked immunosorbent assay (ELISA) using a DuoSet kit (R&D Systems®, Minneapolis, MN, USA) [23].

#### Chemokine (C-X-C motif) Receptor 2 (CXCR2), G protein-coupled receptor kinase 2 (GRK2), ICAM and Chemokine (C-X-Cmotif) ligand 1 (CXCL-1) expression

Six and twenty four hours after surgery, mice were sacrificed; samples of lung and cells of peritoneal cavity were collected. The total RNA was extracted with TRIzol (Invitrogen, Carlsbad, CA, USA). The amount of total RNA was determined spectrophotometrically (Nanoview,GE, Pittsburgh, PA, USA) at 260 nm, and RNA integrity was confirmed by electrophoresis on 1% agarose gels and staining with 0.1 mg/L ethidium bromide. mRNA analysis was performed using a (Life Technologies, Grand Island, NY, USA) Real-time PCR with a SYBR-green fluorescence system. The sequences of the primer pairs were as follows: G protein-coupled receptor kinase 2 (GRK2) forward 5′-ccctctcaccatctctgagc-3′; GRK2 reverse 5′-cggttggggaacaagtagaa-3′; Chemokine (C-X-C) Receptor 2 (CXCR2) forward 5′-tctgctacgggttcacactg-3′; CXCR2 reverse 5′-ggaggaagccaagaatctcc-3′; ICAM-1 forward 5′-cgaaggtggttcttctgagc-3′; ICAM-1 reverse 5′-gtctgctgagacccctcttg-3′; CXCL-1 forward 5′-tgttgtgcgaaaagaagtgc-3′; CXCL-1 reverse 5′-cgagacgagaccaggagaaa-3′. As housekepping we used β*2*M. The sequence of the primer pairs of β*2*M was β*2*M forward 5′-CATGGCTCGCTCGGTGACC-3′; β*2*M reverse 5′-AATGTGAGGCGGGTGGAACTG-3′. To validate and standardize the method, we used positive and negative known control samples for all genes, including the housekepping. The method was procedure as describe previously [25].

#### Nitrite

Tissue (lung) nitrite levels were measured by means of the classic Griess method as described previously [26]. We used 50 µL of the sample, that were prepared as describe above in tissue preparation, to measured nitrite at 540 nm absorbance by using 50 µl of reaction with Griess reagent (sulfanilamide and naphthalene–ethylene diamine dihydrochloride). We wait 10 min at room temperature before read. Amounts of nitrite in the tissue were estimated by a standard curve.

#### Thiobarbituric Acid-Reactive (Tbars)

Thiobarbituric acid-reactive formation was used to quantify the lipid peroxidation in tissues and measured as thiobarbituric acid-reactive as described previously [27]. Tissues were homogenized (100 mg/mL) in 1.15% KCl buffer. A total of 100 µL of the homogenates were then added to a reaction mixture consisting of 750 µL of 0.8% thiobarbituric acid, 100 µL of 8.1% sodium dodecyl sulfate (SDS), 750 µL of 20% acetic acid (pH 3.5), and 300 µL of distilled water. The mixture was then heated at 90°C for 60 min. After cooling at 4°C, the samples were cleared by centrifugation (10,000 *g*, 10 min) and their absorbance was measured at 532 nm, using 1,1,3,3-tetramethoxypropane as an external standard. The level of lipid peroxides was expressed as µmol malondealdeide/mg of protein.

### Peritoneal neutrophils count

For the purpose of cells counting as described previously [28], 28 mice (7 CLP, 7 CLP-H, 7 CLP-S and 7 Basal) were used. After 6 and 24 h, the surviving animals (n = 7/group) were anesthetized and euthanized then peritoneal lavage was obtained in sterile physiological saline solution. The total cells were counted using a Handheld automated cell counter (Millipore® - Billerica, MA, USA). The quantification was performed using a cytocentrifuge; slides were prepared by centrifugation of each sample at 900 *g* for 6 min (Cytospin 2, Shandon Scientific, Pittsburgh, PA). These slides were stained by Diff Quick stain, and differential counts of at least 300 cells were made according to standard morphologic criteria. The result is expressed in cell x10^4^/peritoneum.

### Myeloperoxidase assay

For myeloperoxidase assay we procedure as described previously [27]. Tissues were homogenized (50 mg/ml) in 0.5% hexadecyltrimethylammonium bromide in 10 mM 3-*N*-orpholinopropanesulfonic acid (MOPS) and centrifuged at 15,000 *g* for 40 min. The suspension was then sonicated three times for 30s. An aliquot of supernatant was mixed with a solution of 1.6 mM tetramethylbenzidine and 1 mM hydrogen peroxide. Activity was measured spectrophotometrically as the change in absorbance at 650 nm at 37°C, using a Spectramax microplate reader. Results are expressed as milliunits of myeloperoxidase (MPO) activity per milligram of protein, which were determined with the Bradford assay.

### Immunochemistry

For immunochemistry we procedure as described previously [26]. In the lung, paraffin sections (3 μm) were deparaffinized in xylene and then rehydrated in decreasing concentrations (100%, 95%, and 75%) of ethanol followed by a 10-min incubation in PBS (pH 7.4). Sections were treated with 0.3% hydrogen peroxide for 15 min to block endogenous peroxidase activity and they were then rinsed briefly in PBS. After blocking for nonspecific sites, slides were incubated in a humid chamber overnight at 4°C with the primary ICAM-1 antibody (Santa cruz®, CA, USA). After washing with PBS, slides were incubated following extensive washing (5×5 min) with PBS, immunoreactivity was detected with a biotinylated goat anti-rabbit secondary antibody and the avidin-biotin-peroxidase complex (ABC) both supplied in the Vector Elite kit (Vector Laboratories, Burlingame, CA). Diaminobenzidine (DAB) was used as the chromogen, and the slides were counterstained with hematoxylin.

### Survival rate

CLP was induced in mice, and 30 min later treated with hypertonic saline 7.5% or normal saline solution 0.9%, as described above. We analyzed the survival rate for 168h (7 days). Each group (CLP-C, CLP-S and CLP-H) was composed by 30 animals. After recovery from anesthesia, mice were monitored three times daily. Moribund mice were euthanized by CO_2_ inhalation and cervical dislocation.

### Statistical Analysis

All values were expressed as mean±standard error of the mean (SEM). For the biochemical measurements, the means from the experimental groups were compared by analysis of variance (ANOVA), and Tukey test was used as post hoc test to compare individual groups. To compare the survival curves among different groups of treatment, log rank test was used. These summary measures all represent ‘time to event measures’ and were analyzed using Kaplan Meier survival fit analyses and the Logrank test for statistical significance. This test investigates the null hypothesis that the Kaplan Meier curves for all groups are identical (i.e. that the treatment did not change the time taken to reach the event being analysed). Low P-values are therefore indicative of differences between curves that did not occur due to chance. Statistical significance was assigned to *p*<0.05.

## Results

### Neutrophil infiltration

**Figure 1:**
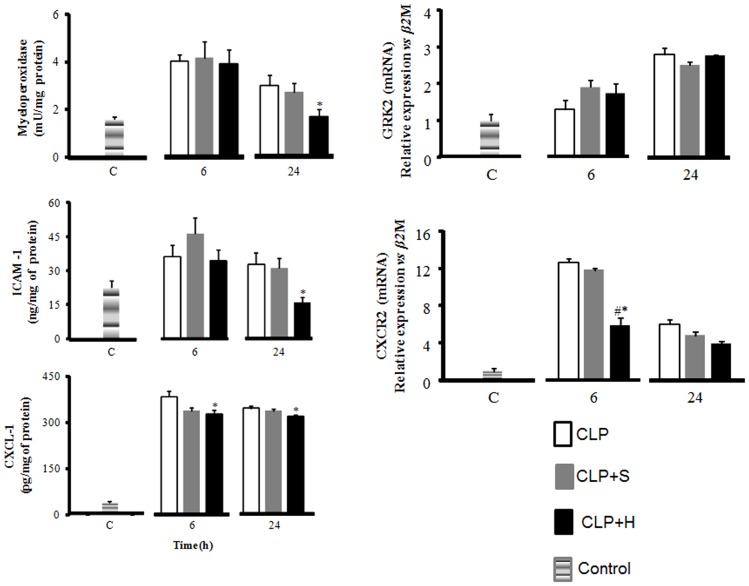
Neutrophil Infiltration in lung. CLP was induced in mice (n = 8 in each group), there were three groups: 1- only CLP (white); 2- 30 min later treated with hypertonic saline solution 7.5% (CLP+H) (black) or 3- treated with normal saline solution 0.9% (CLP+S) (gray). Forth group (C) was used to indicate basal levels. Samples from lung were harvested after 6h and 24h. CLP induced a significant increase in MPO, most notably in lung. Hypertonic solution treatment exhibited a significant reduction in MPO of lung (a). The adhesion molecule ICAM-1 increased after CLP procedure, and hypertonic saline solution presented a significant reduction in the amount of this molecule in lung (b). CXCL-1 presented an important increase after CLP, and hypertonic solution treatment reduced the amount of CXCL-1 in lung (c). Neither groups nor period show any difference in expression of GRK2 (d). CXCR2 expression was increased after CLP procedure and treatment with normal saline, and hypertonic saline solution reduces this expression (e). Data shown represent mean ± SEM. * p<0.05 indicates a significant difference with CLP control; # p<0.05 indicates a significant difference with CLP-S.

We used myeloperoxidase quantification to measure neutrophil infiltration in lung. CLP produced an increase in neutrophil amount in the lung at the period of 6h and 24h post sepsis. Normal saline treatment did not reduce at any time period the amount of neutrophil in the tissue. On the other hand, hypertonic was able in reduce neutrophil infiltration at 24h periods compared to CLP-C group and normal saline group (Figure 1a).

### ICAM e VCAM measurement

We also measured the amount of ICAM-1 (Figure 1b) and VCAM-1 (data not shown) in lung. CLP induced an increase of ICAM-1 amount in the lung. Normal saline group presented a similar profile of ICAM-1 at 6 and 24h periods. Interesting hypertonic produced a progressive decrease of ICAM-1 amount in the lung with a significant decrease at 24h (Figure 1b). CLP did not induce any change in VCAM-1 amount in lung. In addition, the treatments with normal saline or hypertonic solution infusion also did not induce any alteration in lung levels of VCAM-1 in all groups at 6h and 24h periods (data not shown).

### CXCL-1

The data of CXCL-1 in lung of CLP and the groups treated with normal saline or hypertonic saline solution are presented in the Figure 1c. CLP induced an important increase of CXCL-1 amount in the lung matching neutrophil infiltration. CLP and CLP normal saline groups did not show a significant decrease of CXCL-1 at 24h periods. Interesting hypertonic produced a significant decrease of CXCL-1 amount in the lung (Figure 1c).

### Chemokine (C-X-C) Receptor 2 (CXCR2) and G protein-coupled receptor kinase 2 (GRK2) expression in lung

We measured the expression of GRK2 and CXCR2 in lung (Figure 1d and e, respectively). Neither groups and nor period show any difference in expression of GRK2. CLP and CLP+S groups induced an increase of CXCR2 expression in relation to CLP+H that reduces this expression at 6h. Even at 24h we didn’t found any statistical difference, the group CLP+H has a tendency to reduce the levels of CXCR2 in comparison to CLP group.

### ICAM-1 in the tissues

**Figure 2:**
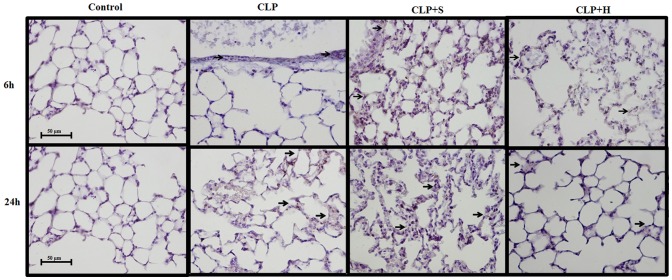
ICAM-1 staining in lung. CLP was induced in mice (n = 8 in each group), there were three groups: 1- only CLP; 2- 30 min later treated with hypertonic saline 7.5% (CLP+H) or 3- treated with normal saline solution 0.9% (CLP+S). Forth group (C) was used to indicate basal levels. Samples from lung (a) were harvested after 6, 12 and 24 h. There was a positive staining for ICAM-1 in CLP animals, showing the presence of inflammation activating molecules for neutrophil recruitment. There was no difference in the intensity of ICAM-1 staining between CLP and CLP treated with normal saline. Hypertonic solution reduced ICAM-1 activation in CLP mice.

The immunohistochemistry stain for ICAM-1 (Figure 2) shows clearly the presence of ICAM-1. The ICAM at CLP group was present in capillary close to alveolus in lung, and hypertonic present lower amount of staining (Figure 2).

### Cytokines measurements

We measured the amount of TNF-α, IL-6 e IL-10, in lung. These cytokines provide the predominant inflammatory profiles post CLP in the tissues. In addition, we can analyze the effect of normal saline or hypertonic infusion in the inflammation response.

#### Tumor Necrosis Factor-α

**Figure 3:**
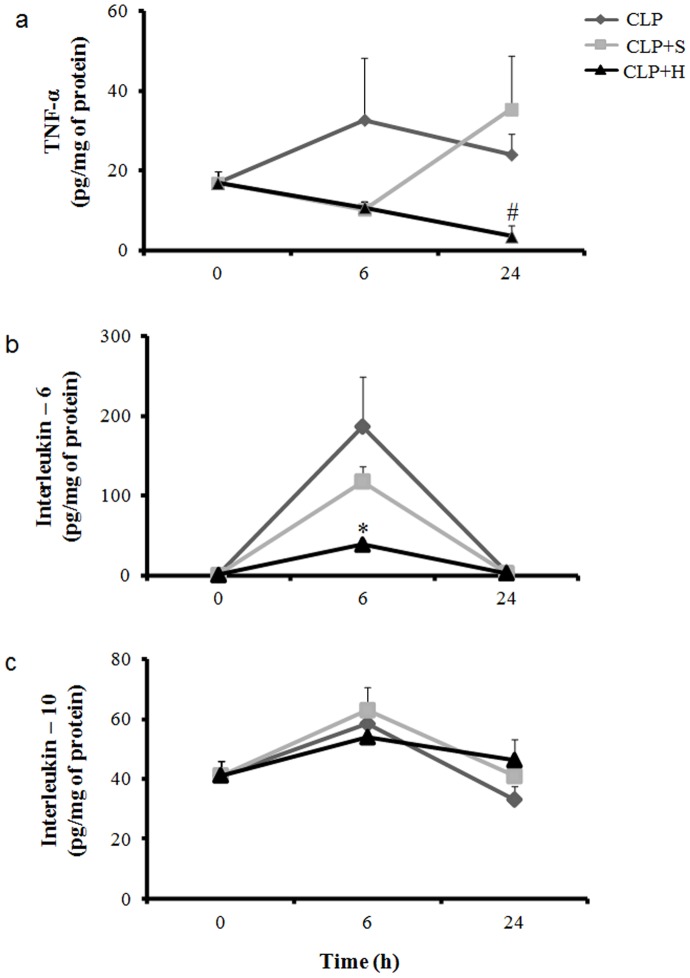
Interleukin measurements in lung. CLP was induced in mice (n = 8 in each group), there were three groups: 1- only CLP (white); 2- 30 min later treated with hypertonic saline solution 7.5% (CLP+H) (black) or 3- treated with normal saline solution 0.9% (CLP+S) (gray). Tissue amount of selected pro- and anti-inflammatory cytokines were measured 6h and 24 h after sepsis. CLP induced significant increase in cytokines compared to basal levels. Hypertonic solution had significantly reduced TNF-α (a) and IL-6 (b) in lung, while IL-10 (c) was increased at 6h in lung. Data shown represent mean ± SEM. * p<0.05 indicates a significant difference with CLP control; # p<0.05 indicates a significant difference with CLP-S

The lung content of TNF increased after CLP, however, there was an interesting fact, normal saline treatment produced higher amount of TNF at 24h compared to CLP control group. Small volume and hypertonic on the other hand, reduced TNF amount in the lung in all periods studied (Figure 3a).

#### Interleukin-6

In lung we observed an important increase in the IL-6 amount post sepsis in the groups of CLP-C and CLP-S compared to basal quantification. The infusion of normal saline did not produce any change in IL-6 compared to CLP control group. CLP-H group presented a significant reduction at 6h compared to CLP control (Figure 3b).

#### Interleukin-10

There was an increase in IL-10 post CLP in lung compared to basal condition. The treatments with normal saline or hypertonic solution did not reduced IL-10 amount during the 24 hours (Figure 3c).

### Oxidative stress

#### Malondealdehyde

**Figure 4:**
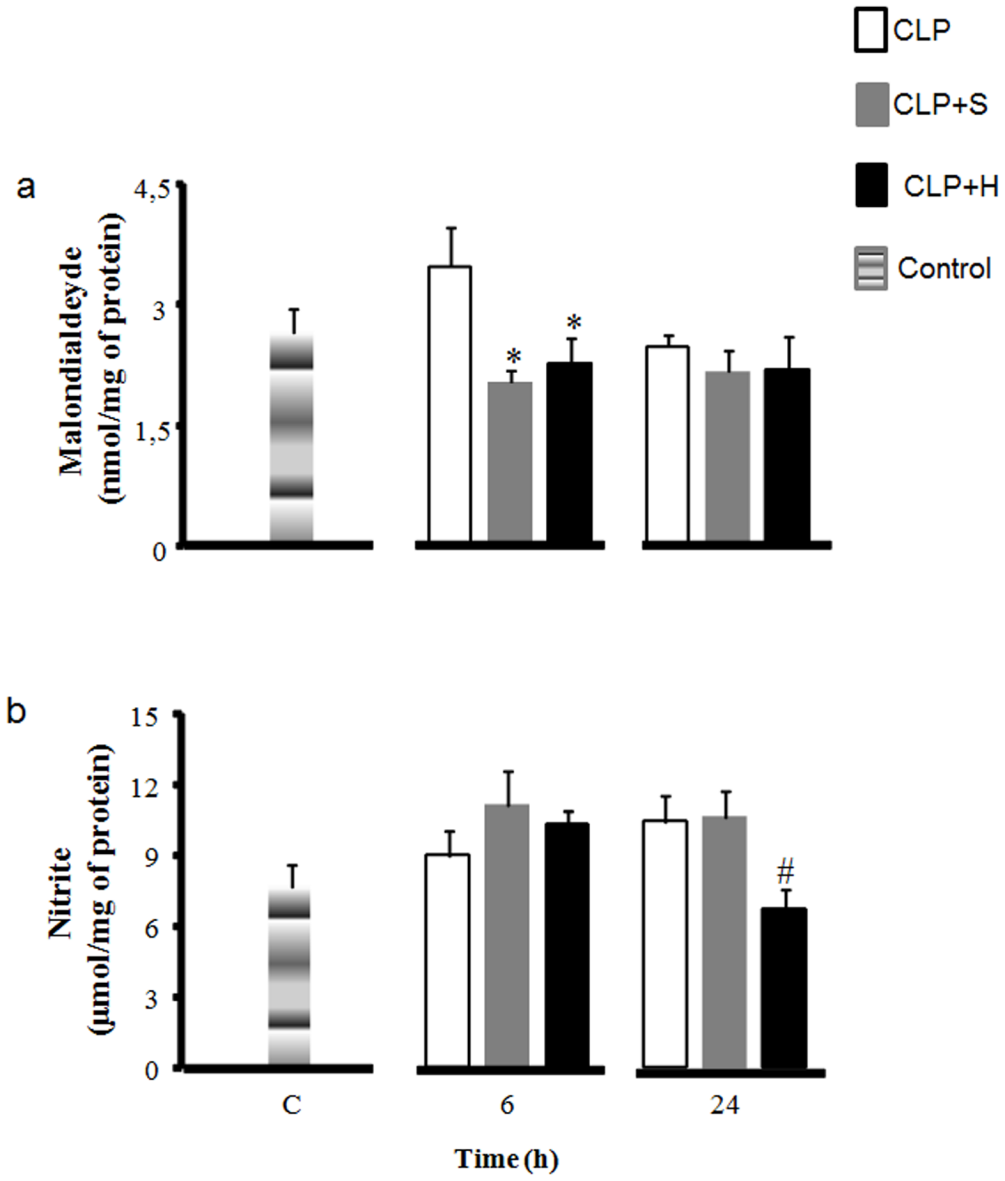
Lipid peroxidation and oxidative stress. CLP was induced in mice (n = 8 in each group), there were three groups: 1- only CLP (white); 2- 30 min later treated with hypertonic saline solution 7.5% (CLP+H) (black) or 3- treated with normal saline solution 0.9% (CLP+S) (gray). Forth group (C) was used to indicate basal levels. Sample from lung were harvested after 6h and 24h for nitrite and lipid peroxidation measurements. CLP induced significant increase in MDA content in the tissue. The animals treated with normal saline or hypertonic solution presented a reduction in MDA content in lung (a). There was increased NO production after sepsis, and there was no difference in this respect between all groups (b). Data shown represent mean ± SEM. * p<0.05 indicates a significant difference with CLP control; # p<0.05 indicates a significant difference with CLP-S.

In lung, the levels of MDA were high in CLP control group compared to the groups that receive volume replacement at 6h period (Figure 4a). Both treatments were effective in reducing MDA production.

#### Nitrite

Our data of nitric oxide indicates an increase after CLP procedure in the lung. The use of volume infusion with normal saline did not produce any change in the amount of NO. The infusion of hypertonic solution showed an important reduction of nitric oxide at 24h period (Figure 4b).

### Chemotaxia and infiltration of neutrophil in peritoneum cavity

**Figure 5:**
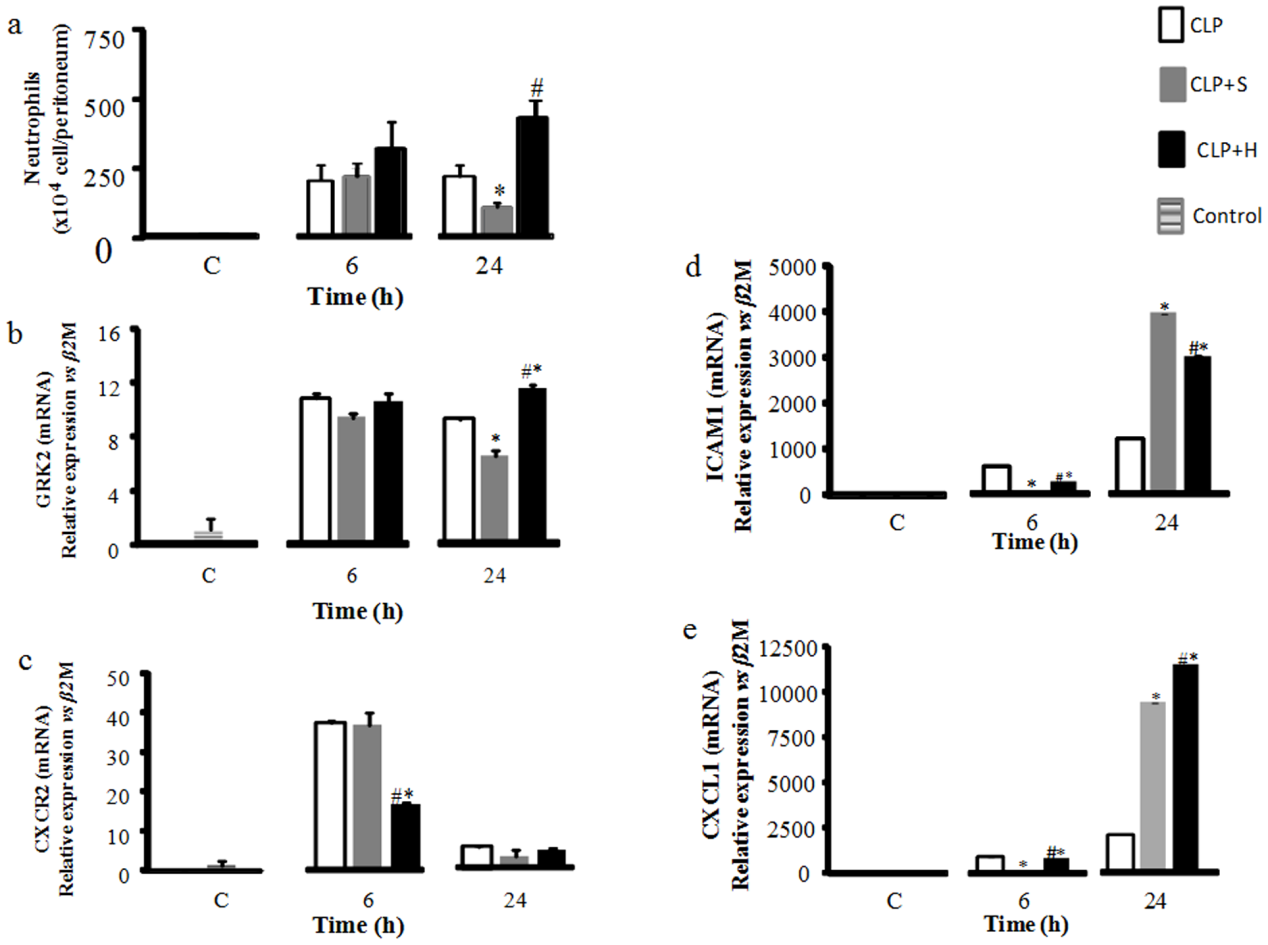
Total leukocyte counts peritoneal lavage fluid (PLF). CLP was induced in mice (n = 8 in each group), there were three groups: 1- only CLP (white); 2- 30 min later treated with hypertonic saline solution 7.5% (CLP+H) (black) or 3- treated with normal saline solution 0.9% (CLP+S) (gray). Forth group (C) was used to indicate basal levels. A neutrophil count was determined in PLF obtained 6, 12 and 24 hours after CLP. Sepsis induced a significant increase in the neutrophil amount in peritoneal cavity. Normal saline reduced the neutrophil at 24h, while hypertonic increased the neutrophil in this period (a). GRK2 expression increased in CLP and CLP+S group in relation to basal levels, and the expression in CLP+H was increased in relation to CLP and CLP+S (b). CXCR2 expression was increased after CLP procedure and treatment with normal saline, but the expression of CXCR2 was decreased in CLP+H group (c). ICAM-1 expression in group CLP and CLP+H were increased in relation to CLP+S group (d). CXCL-1 expression in the groups that receive treatment reduces in relation to CLP group, interesting that the CLP+H group was higher than CLP+S (e). Data shown represent mean ± SEM. * p<0.05 indicates a significant difference with CLP control; # p<0.05 indicates a significant difference with CLP-S.

We performed a peritoneal lavage and posterior neutrophil counting in order to analyze the effect of hypertonic solution on neutrophil migration to the infectious site. We shown in the figure 5a that post CLP, the group without any treatment presented a progressive increase in neutrophil per cavity. Normal saline infusion presented an inhibition of the protective neutrophil migration to the infectious site at 24h period. On the other hand, our data show that hypertonic solution increase neutrophil migration in the first 24h with a benefit in the clinical evolution.

We also measured GRK2, CXCR2, ICAM and CXCL-1 to analyze the infiltration of neutrofhil in peritoneal cavity; those data are presented in the Figure 5b to e. The expression of GRK2 in CLP and CLP+S group was rise in relation to basal levels, and the group treated with hypertonic saline solution was increased in relation to CLP and CLP+S groups at 24h. The expression of CXCR2 in CLP+H was decreased in relation to CLP and CLP+S group at 6h. The CLP and CLP+H increased the expression of ICAM-1, whereas the CLP+S reduce this expression at 6h. At 24h the group that receive normal saline treatment show a rise of ICAM-1 expression. Whereas the CLP and CLP+H reduced the expression, even the CLP+H show a increased in relation to CLP group.

The CXCL1 expression in the groups that receive treatment reduces in relation to CLP group, interesting that the CLP+H group was higher than CLP+S at 6h and 24h.

### Survival rate

**Figure 6:**
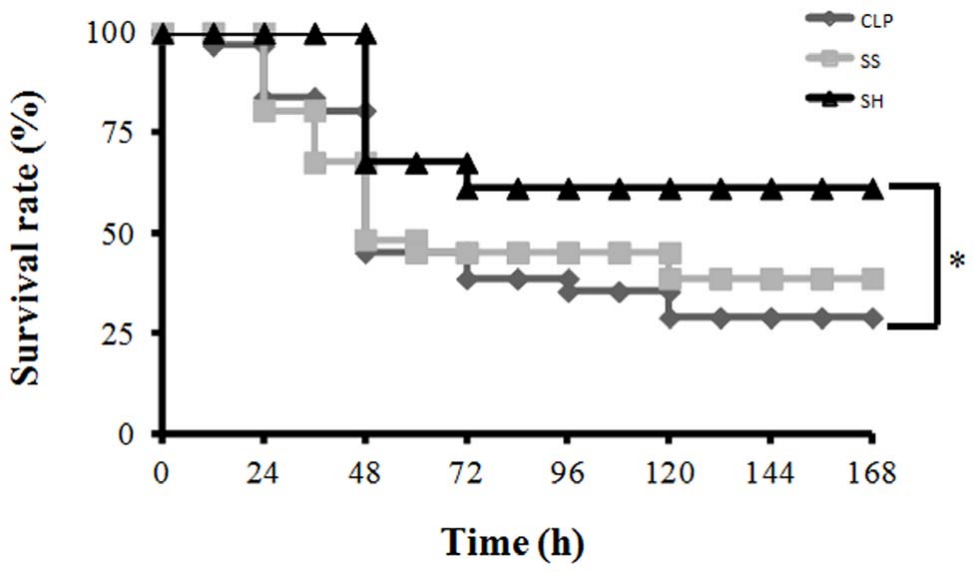
Survival experiments in CLP: the role of hypertonic saline solution treatment. CLP was induced in mice, three groups were analyzed: 1- CLP without any treatment; 2- CLP with normal saline 0.9% treatment; and 3- CLP treated with hypertonic saline solution 7.5% (n = 30 in each group). The animals were observed for 7 days, and mortality was recorded every 12h. We observed that the group treated with hypertonic solution had a better survival rate (60%), in comparison with CLP-S group (46.66%) and C group (33.33%) in the end of the seventh day. No change in mortality was observed after Day 5 in either experimental group. *p<0.05, Log-Rank test.

We observed that the group treated with hypertonic solution had a better survival rate (60%), in comparison with CLP-S group (46.66%) and C group (33.33%) in the end of the sixth day (Figure 6).

## Discussion

Septic patients present organ damage and dysfunction; the therapeutic interventions have not changed significantly the mortality rate. Specific therapeutic interventions that can reduce the high mortality rates are needed [29]. The cecal ligature and puncture model closely resemble the course of sepsis observed in patients, characterized by an early hyperdynamic, hypermetabolic state [30]. In our study the animals submitted to CLP received intravascular fluid replacement with normal saline or hypertonic saline solutions. Since the 1980’s, the HSS has been extensively studied in experiments that show an improvement in hemodynamic, using peripheral vein infusion of a dose of 4 ml/kg [17]–[20]. The volume infused in regular saline group was 34 ml/Kg, the reason for this amount of saline used is to reach the same load of NaCl infused with hypertonic solution. Infusing the same load of *sodium* is important because its solute determines the extracellular volume. In addition, the volume calculated for saline is almost the same proposed in the literature for treat septic shock (River, sepsis surviving campaign). Our results shown that animals submitted to polymicrobial sepsis treated with hypertonic solution have a reduced mortality rate. The data demonstrated reduction in proinflammatory cytokines, chemokines, ICAM-1, CXCL-1, CXCR-2 and neutrophil infiltration in lung. The peritoneal cavity has a reduction of ICAM-1, CXCR-2 and GRK-2 in the hypertonic group. However, the group of hypertonic saline treatment presented increased elevation of chemokine CXCL-1 and elevation in the peritoneal cavity neutrophil amount preserving a high capacity of fighting bacteria in the infectious site. The physiopathology of sepsis includes increased vascular permeability with consequent volume extravasation to interstitial space and hemodynamic instability [31]. Precocious volume infusion is a necessary therapeutic intervention in patients with septic shock [32]. However, large volume reposition can induce or aggravate pulmonary interstitial edema and increase intra-abdominal pressure [16], [33]. Lung water accumulation exacerbates respiratory failure and makes mechanical ventilation necessary. An increment in abdominal pressure reduces venous return decreasing cardiac output, with the consequences of lower tissue perfusion and organ damage. These data demonstrate the relevance of small volumes solutions infusion as an alternative to treat septic patients.

Neutrophil are the first line of defense against infection challenge and key part of the innate immunity [34]. Their arsenal of proteases and reactive oxygen species make neutrophil very efficient for killing bacteria, and at the same time may cause host tissues damage [35], [36]. CLP group presented in the lung an increased neutrophil infiltration along the 24 hours period post sepsis. The lung is a bystander distant target organ in peritoneal sepsis disease that is frequently damaged by the inflammation. Normal saline was ineffective in control neutrophil infiltration in lung. On the other hand, the treatment with hypertonic solution presented a significant reduction in neutrophil infiltration along 24h period. Neutrophil recruitment to the site of inflammation is mediated by adhesion molecules such as, selectins, integrins, immunoglobulin superfamily, chemokines and G-protein coupled receptors [37]. ICAM-1 is an important adhesion molecule that participates on neutrophil recruitment. There is a mechanistic explanation for the action of hypertonic solution that is through adhesion molecules in sepsis, reducing neutrophil infiltration and organ damage. CLP-H group presented a time course of ICAM-1 in the lung parallel to neutrophil infiltration and at 24h the reduction was significant in both ICAM-1 and neutrophil. In addition, there was lower amount of proinflammatory cytokines (TNF-α, IL-6) in the lung which permits to reduced ICAM activation and consequent less neutrophil. ICAM-1 and VCAM-1 are present constitutively on endothelial cells *in vitro* and *in vivo*, the expression of ICAM-1 can be augmented by a variety of inflammatory mediators, such as tumor necrosis factor α (TNF-α) and endotoxin [38]. On the other hand, there was a reduction of CXCL-1 and CXCR-2 amount in lung tissue of animals treated with hypertonic solution in all periods measured. Taken together these data can explain the mechanistic action of hypertonic by increasing GRK-2 which reduces CXCR-2 in endothelial cells, also hypertonic solution reduced expression of ICAM_1 and CXCL-1.

The final hypertonic effect presented a lower inflammation in lung and in addition to this, lower amount of superoxide and nitric oxide. The superoxide production was reduced early by hypertonic solution treatment, at 6h and was kept at the baseline. On the other hand, nitric oxide was affected late by hypertonic treatment. These results are consistent with previous results found in experimental pancreatitis treated with hypertonic solution. Nitric oxide has beneficial effects, and high levels of superoxide and nitric oxide produce peroxinitrite, that is more reactive and toxic to cell [29]. In that way hypertonic treatment reducing preferentially superoxide potentiate the nitric oxide beneficial [26].

Interestingly hypertonic saline *increased* neutrophil amount in the, infectious site, peritoneal cavity. The literature has shown as a determinant factor in the sepsis survival the quantity of neutrophil in the infectious focus [37], [39]. The phagocytes are necessary in order to eliminate the invaders bacteria. After 24 hours of cecal ligation and puncture, animals that received HSS had fewer bacteria in serum, lower formation of abscesses in liver and lungs, and less pulmonary and hepatic injury [40]. However, neutrophil migration to bystander organs, as for instance lung, causes a cost without benefit. Our data are in agreement to Jones et al, their study showed that an inhibition of neutrophil migration to the site of infection as consequence develop an increase remote organ neutrophil sequestration and injury [41]. Interesting hypertonic solution reduced neutrophil infiltration in lung, through its action on ICAM-1, CXCR-2 and CXCL-1. The partial and less potent anti-inflammatory effect of hypertonic saline was sufficient for bystander organs, where the inflammation is not the focus. On the other hand, in the infectious focus predominates the hemodynamic effect of hypertonic solution increasing perfusion and neutrophil delivery [20]. In that way CXCR-2 and ICAM-1, a neutrophil receptor and an endothelial adhesion molecule were reduced by hypertonic solution as in the lung. However, the action of hypertonic solution was a favorable increase in peritoneal macrophage CXCL-1 release improving neutrophil migration to the infectious site.

The survival was 33% in CLP-C with no treatment, on the other hand volume expansion showed a reduction in mortality. The animals receiving normal saline presented a 47% survival and the hypertonic group showed a significant higher survival (60%). Following the initial microbial interaction there is widespread activation of innate immune response, the function of host defense is the elimination of the invading organism or destruction of foreign tissue. Inflammation is the price paid for an effective defense, that consist in releasing pro-inflammatory cytokines, such as TNF-α, IL-1, IL-6 [42], [43], however, an excessive activation can lead to multiple organ dysfunction and death [1]. The subsequent effect, and sometimes concomitant, is the release of anti-inflammatory mediators, such as a few cytokines (IL-10, IL-4, IL-13, transforming growth factor β (TGF-β) [43]. Hypertonic saline solution did not reduce completely pro-inflammatory pathways, modulating the balance in favor to the anti-inflammatory cytokines compared to pro-inflammatory cytokines, maintaining the ability to fight bacteria efficiently and at same time reducing organ damage [40]. Our data corroborate the recent study in septic patients that showed benefits of hypertonic solution in the treatment compared to normal saline [21].

## Conclusion

Hypertonic saline (NaCl7.5%) improves the protection against infection by increasing the infiltration of neutrophils in the peritoneum, i.e. the focus of infection, however, at the same time on the distant target organ decreases the infiltration lung neutrophils, these two actions associated may explain the protective effect of hypertonic saline solution. Finally, hypertonic solution can reduce the mortality of septic animals [12].
